# Supplementary material for: MetaRibo-Seq measures translation in microbiomes
Source: Nat Commun. 2020 Jun 29;11:3268. doi: 10.1038/s41467-020-17081-z (PMC7324362; doi:10.1038/s41467-020-17081-z)
Supplement: Supplementary file 10 — Supplementary Data 7 [file 41467_2020_17081_MOESM10_ESM.zip › File2/Confidence_High_Taxonomy/199759_out.krona.html]

Javascript must be enabled to view this page.

members
magnitude
magnitudeUnassigned
count
unassigned
taxon
rank

199759\_out

13

13
2
superkingdom

13
1239
phylum

13
186801
class

order
186802
13


SRS011084\_contig\_number\_contig-100\_3675.189403SRS011239\_contig\_number\_contig-100\_12485.100386SRS016018\_contig\_number\_6422SRS016203\_contig\_number\_contig-100\_39294.39294SRS020869\_contig\_number\_36610SRS023914\_contig\_number\_4239SRS056273\_contig\_number\_contig-100\_4127.169566SRS075078\_contig\_number\_26684SRS076929\_contig\_number\_9057SRS098571\_contig\_number\_65561SRS1041145\_contig\_number\_34376SRS104400\_contig\_number\_52628SRS1055069\_contig\_number\_contig-100\_1268.1269
1898207
species
13
